# Percutaneous Endoscopic Transforaminal Lumbar Interbody Fusion for the Treatment of Lumbar Spinal Stenosis: Preliminary Report of Seven Cases with 12-Month Follow-Up

**DOI:** 10.1155/2019/3091459

**Published:** 2019-03-24

**Authors:** Jincai Yang, Chang Liu, Yong Hai, Peng Yin, Lijin Zhou, Yaoshen Zhang, Aixing Pan, Yangpu Zhang, Liming Zhang, Yi Ding, Chunyang Xu

**Affiliations:** Department of Orthopedic Surgery, Beijing Chao-Yang Hospital, Capital Medical University, 100020, China

## Abstract

**Purpose:**

The objective of this study was to investigate the preliminary effectiveness of percutaneous endoscopic transforaminal lumbar interbody fusion (PE-TLIF) for the treatment of lumbar spinal stenosis (LSS).

**Methods:**

From September 2016 to June 2017, a series of seven patients consisting of six females and one male with an average age of 55.25 years (range 43–77 years) who were diagnosed with LSS were involved in this study. All patients were treated by PE-TLIF. During perioperative and follow-up period, demographic data, operation time, intraoperative blood loss, Visual Analogue Scale (VAS), Oswestry Disability Index (ODI), and modified MacNab criteria were evaluated and perioperative complications were documented.

**Results:**

All patients were followed up for more than 12 months, with an average follow-up time of 15 (range 12-21) months. The mean VAS of back pain was 7.43 (range 6-8) preoperatively and 0.86 (range 0-2) at the final follow-up. The mean VAS of leg pain was 6.14 (range 4-9) preoperatively and 0.71 (range 0-1) at the final follow-up. The mean ODI was 53.57% (range 38%-63%) preoperatively and 15.57% (range 5%-26%) at the final follow-up. In three-month follow-up, continuous bone trabeculae bridging between intervertebral bodies was seen in 3 cases, and the remaining 4 cases could identify continuous bone trabeculae bridging at 6-month follow-up, reaching the standard of spinal intervertebral fusion. At the final follow-up, 4 patients were rated as excellent (4/7) and 3 patients were rated as good (3/7) according to the modified MacNab criteria.

**Conclusions:**

Our study suggested that percutaneous endoscopic transforaminal lumbar interbody fusion could acquire satisfactory treatment effects for the patients with lumbar spinal stenosis, even for the patient who could not afford general anesthesia.

## 1. Introduction

Low back pain is the major worldwide pathology of disability which gives rise to an increasing social burden among the expanding and ageing population [1]. It is reported that 50% of elder patients suffer from lumbar degenerative diseases accompanied by low back pain [2]. LSS is the main type of lumbar degenerative diseases [3] and open surgery including laminectomy and lumbar fusion has become the standard procedure for the treatment of LSS since 1990s [4, 5]. Traditional operations can acquire good curative effect, whereas high complication rates were reported owing to severe paraspinal iatrogenic damage and potential risks of nerve root injury [6]. In 2002, Khoo and Foley firstly reported MIS-TLIF (minimally invasive TLIF) [7] and advantages of MIS-TLIF included the following aspects: reduced paraspinous muscle injury, minimized perioperative blood loss, quicker recovery time, and reduced risk of infection at surgical sites [8, 9]. However, necessary resection of facet joint and lamina may pose a threat to postoperative symptomatic release and lumbar instability [9, 10]. Jacob et al. reported a systematic review of 5454 MIS-TLIF patients and complication rate was 19.2% among which 20.16% were paresthesia, 2.22% transient, and 1.01% permanent nerve damage [11]. Therefore, a minimally invasive procedure with well-designed paraspinous muscle preservation and nerve protection may be necessary.

In 1997, the Yeung Endoscopic Spine System (YESS) developed by Yeung was approved by FDA [12]. Since then, percutaneous endoscopic lumbar discectomy (PELD) has progressed rapidly [13, 14]. The booming PELD technique inspired spine surgeon to perform endoscopic lumbar interbody fusion. In 2012, Said et al. reported 60 cases of endoscopic transforaminal decompression, interbody fusion, and percutaneous pedicle screw implantation for the treatment of lumbar degenerative diseases with 59.6% solid fusion and 36.2% stable fixation, but complication rate was up to 20% [15]. Jacquet et al. reported 57 endoscopic lumbar interbody fusion cases and achieved good clinical result and immediate standing and walking [16]. However, the study also reported that complication rate was up to 36% and the authors began to depend on technical improvements. A more recent study presented percutaneous transforaminal endoscopic lumbar interbody fusion with expandable spacers (B-Twin) for 18 patients, while radiological results showed disc space subsidence in all patients and breakage of implant limbs in 5 patients and revision surgery was performed in 1 patient [17]. The authors believed modifications in implant design were necessary improvements.

With the advancement of endoscopic spine technique and further understanding of minimally invasive ideas, we conducted the research and development of PE-TLIF with guided superior articular process (SAP) resection device, parallel expandable cage, and medical equipment of larger diameter for the treatment of LSS.

The main purpose of this study was to share our preliminary clinical experiences and results of PE-TLIF in the treatment of LSS at a single center with a minimum of 12-month follow-up.

## 2. Materials and Methods

This study was approved by institutional review board (IRB) of Beijing Chao-Yang Hospital. From September 2016 to June 2017, a series of seven patients consisting of six females and one male with an average age of 55.25 years (range 43–77 years) who were diagnosed with LSS were involved in this study. All patients were aware of all possible outcomes of this procedure and signed written consent before operation.

During perioperative and follow-up period, demographic characteristics, comorbidities, surgical level, surgical time, blood loss, time to ambulation, time to discharge, fusion time, and perioperative complications were collected and well documented. We evaluated clinical outcomes using VAS for low back pain and leg pain at their preoperative examination, early postoperative stage, and final follow-up. ODI scores were measured before operation and at last follow-up. Satisfaction of patients was graded into excellent, good, fair, and poor using modified MacNab criteria. Preoperative radiological studies included lumbar spine standing X-rays, computerized tomography (CT), and magnetic resonance imaging (MRI) studies. During follow-up period, X-rays and CT were clear enough to identifying underlying failure and evaluating spinal fusion. Further details were listed Table 1.

## 3. Surgical Technique

**Figure 1:**
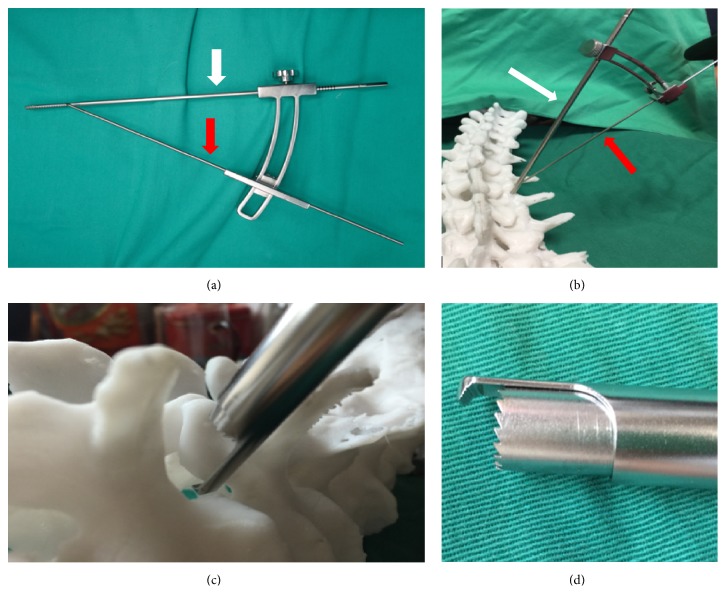
Guided SAP resection device and its schematic working picture, the white arrow pointing to the primary guide pin and the red arrow pointing to the secondary guide pin and the arch being between them. (a) Holistic view of guided SAP resection device. (b) Holistic view of schematic working picture. (c) Hook-shaped front of the cannula restricted the depth of incision. (d) Feature view of the hook-shaped front.

**Figure 2:**
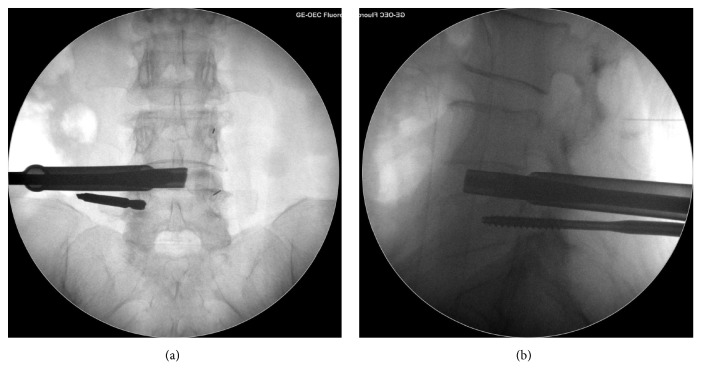
Using fluoroscope to ensure the position of the reamer when preparing endplate. (a) Anteroposterior view of the position of the reamer. (b) Lateral view of the position of the reamer.

**Figure 3:**
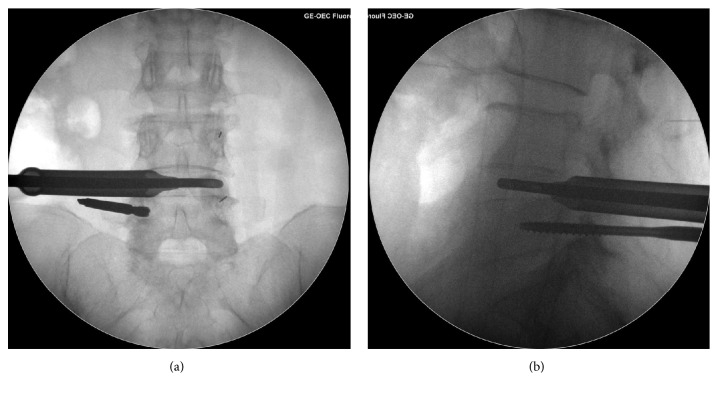
Using fluoroscope to ensure the position of the forceps when preparing endplate. (a) Anteroposterior view of the position of the forceps. (b) Lateral view of the position of the forceps.

**Figure 4:**
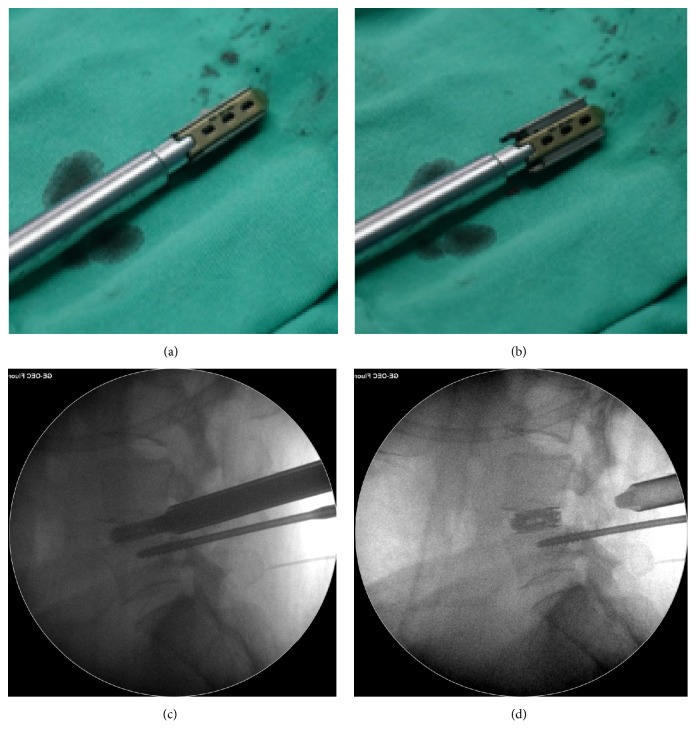
Titanium parallel expandable cage. (a) Holistic view of the cage when unexpanded. (b) Holistic view of the cage when expanded. (c) Unexpanded cage under fluoroscope. (d) Adequately expanded cage under fluoroscope.

The patients were positioned supine on a radiolucent table. It was flexible to select low dose epidural anesthesia combining with local anesthesia or general anesthesia based on physical condition and willingness of patients. Pedicles of two vertebral bodies adjacent to lesion segment were presented by intraoperative C-arm fluoroscope. Then a specially designed SAP guider was installed, containing a primary guide pin, a secondary guide pin, and a connecting arch (see Figure 1(a)). Under the guidance of fluoroscope, the primary guide pins were inserted into pedicles on the symptomatic side. Connecting through the arch, secondary guide pin was placed at SAP. Then dilating and protection cannulas were inserted progressively and the depth of incision was restricted by a hook-shaped front of the cannula so that trepan could reach SAP while protecting soft tissues and nerve (see Figure 1). The majority of SAP was excised and taken out by trepan and the intervertebral foramen was enlarged. With the guidance of a guide rod, working channel was placed through Kambin's triangle. Then the endoscope was connected and the working channel was moved right to the intervertebral disc. Working cannula was rotated to keep the exiting nerve root on a safe status. Under endoscopic monitoring, ligament flavum dissection was performed, and the remaining SAP was removed by endoscopic kerrison or burr drill. Then canal was decompressed and nerve root was released. After confirming that the nerve structures were decompressed, the endoscope was removed and discectomy was conducted through an implantation tube which had an inner diameter of 11.5mm that provided a safe and easy access to intervertebral space for instruments with larger size such as reamers, bone curettes, and forceps. The implantation tube was placed into the intervertebral space with a fork-shaped tip, and these two edges could keep the traversing and existing nerve root protected out of the working channel simultaneously while performing a complete endplate preparation. Disc materials were firstly excised with reamers. For adequate endplate preparation, the reamer was inserted until the tip reached more than 3/4 diameter of the intervertebral space under fluoroscope and reamers with larger size (from 7mm to 11mm) were inserted progressively (see Figure 2). Next, curettes and forceps were entered to remove disc materials and the positions were also checked by fluoroscopy images (see Figure 3). Tactile feedbacks from reamers and curettes could tell approximate size of the instruments that should be used and whether the endplates were reached. After removal of the disc materials, endoscope was installed again to make sure that the cartilaginous endplates were scraped away, intervertebral space was fully prepared, and the appearance of exudation from bone endplate was acceptable. Adequately the endplates were prepared, the endoscope was taken out, and graft bone was implanted through the implantation tube. To ensure a solid fusion, the intervertebral space was packed with bone from excised SAP and iliac bone autograft (or allogeneic bone when necessary) and total mass of bone grafting must be 10 mm^3^ and over. Expandable cage (Shanghai REACH Medical Instrument Co., Ltd, Shanghai, China) was then inserted through the implantation tube (see Figure 4). The spinal canal was checked with endoscope, making sure the nerve root was totally relieved. Primary pins were replaced with guide wires and 4 pedicle screws were implanted into planned positions. Two rods were inserted percutaneously; sequentially the screw-rod attachment was tightened. Sutured skin and the position of instruments were rechecked by a fluoroscope.

## 4. Results

**Figure 5:**
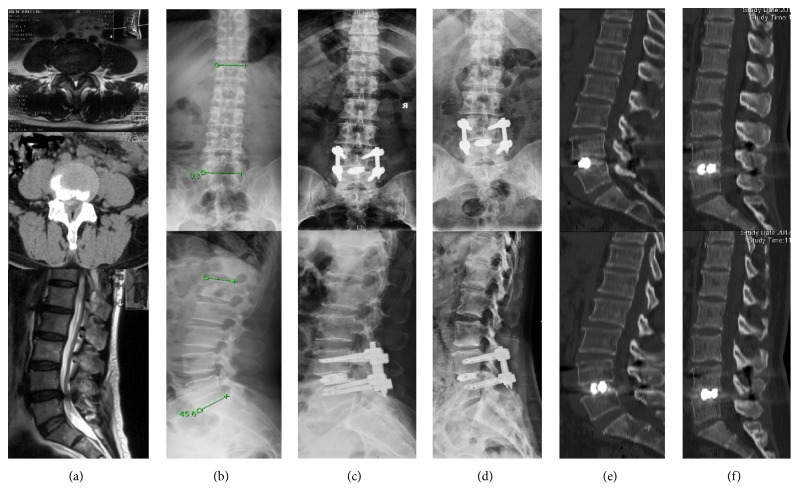
A 57-year-old female patient who had low back pain with right leg pain and numbness for 2 years, intermittent claudication 100m, and was treated by PE-TLIF. (a) Preoperative MRI and CT images showed a moderate lumbar spinal stenosis. (b) Preoperative X-ray image showed no instability. (c) Postoperative X-ray image showed a good implantation position. (d) Six-month follow-up X-ray image showed neither disc space subsidence nor implantation breakage. (e) Postoperative CT scan image. (f) Six-month follow-up CT scan image showed a standard lumbar fusion.

All patients were followed up for more than 12 months, with an average follow-up time of 15 (range 12-21) months. The mean VAS of preoperative back pain was 7.43 (range 6-8) and mean VAS of back pain was 0.86 (range 0-2) at the final follow-up. The mean VAS of preoperative leg pain was 6.14 (range 4-9) and mean VAS of leg pain was 0.71 (range 0-1) at the final follow-up. The mean preoperative ODI was 53.57% (range 38%-63%) and mean ODI was 15.57% (range 5%-26%) at the final follow-up. In three-month follow-up, continuous bone trabeculae bridging between intervertebral bodies was seen in 3 cases, and the remaining 4 cases could identify continuous bone trabeculae bridging at 6-month follow-up, reaching the standard of spinal intervertebral fusion. At the final follow-up, 4 patients were rated as excellent clinical outcomes (4/7) and 3 patients were as good clinical outcomes (3/7) according to the modified MacNab criteria. Further details were listed in Table 2 (see Figure 5).

## 5. Complications

In one case (case 4), the anterior side of intervertebral disc was ruptured during endplate preparation, but no significant blood vessels, nerves, or internal organs were damaged. One patient (case 2) experienced temporary knee tendon hyperreflexia after surgery and recovered within 24 hours after surgery. No damages to the exiting nerve root, traversing nerve root, and dura mater were found.

## 6. Discussion

This is a retrospective study of an innovative minimally invasive spine surgery for the treatment of degenerative LSS. The present study showed that the treatment of degenerative LSS by PE-TLIF achieved satisfactory clinical and radiological results. The function of paraspinal muscle was reserved entirely and elaborately by this treatment, and a solid fusion of involved segment was obtained within three to six months. All the patients could return to work 3 months after operation which generally reduced the burden on individuals, families, and communities. There was no sign of segmental instability, muscle weakness, paresthesia, or cauda equina syndrome by radiographic and clinical examination in all the patients. All the patients were fulfilled with the treatment.

With the rapid development of science and technology, doctors were searching for minimizing injury while acquiring best results. It was said that paraspinal muscle was the key to support extension of the spine, maintain lumbar lordosis, and achieve spinal dynamic stability [6]. The traditional open lumbar interbody fusion operation (TLIF/PLIF) was regarded as standard procedure for the treatment of various degenerative lumbar disorders [4, 5]. However, the significant paraspinal iatrogenic injury caused by prolonged muscle retraction and dissection and stripping of tendinous attachments could not be ignored for resulting in delayed recovery and mobilization due to approach-related muscle trauma during these procedures. Meanwhile application of electrocautery could bring about direct cauterization of blood vessel, muscle tissue, and even unrecognized nerve [6, 18]. It destroyed the blood supply for the muscles and made them weak and difficult to maintain the dynamic stability of the spine. Aiming at reducing direct dissection and surgical trauma to important anatomical structures, MIS-TLIF was first put forward by Foley [7]. Many studies have reported satisfactory clinical results and less complication rates of MIS-TLIF compared with traditional procedures [8, 19, 20]. The Wiltse approach with simple microhooks could reduce muscle detachment to some extent, but its longer operative time inevitably led to muscle retraction and damage for a long time [9]. Meanwhile MIS-TLIF had a steep learning curve, which required accumulation of certain of cases to acquire good knowledge of the technique [10]. The reported critical point of learning curve was 44 cases [19]. Although MIS-TLIF could have desirable results, still high complication rates and steep learning curve made it an unsatisfactory candidate for ideal minimal invasive lumbar interbody fusion procedure.

On the basis of the experiences gained from open spinal procedures and the desire to minimize surgical trauma while obtaining great results, an attempt to perform lumbar interbody fusion with the help of endoscope has emerged with the evolution of the lesser invasive spinal procedures. The approach of PE-TLIF was an improved transforaminal approach, in which the majority of SAP was excised and endoscopic instruments of larger diameter could have access to this enlarged foramen to make sure decompression is completed and endplates are fully prepared. As one of our surgical focuses was nerve protection, surgical procedure and key points were centered on how to prevent nerve structure from injury. The design of guided SAP resection was based on the relative constant anatomy relation between SAP and pedicles in lumbar spine to remove SAP without nerve damage (see Figure 1). The depth of incision was restricted by a hook-shaped front of the cannula used for SAP resection which kept exiting nerve root and dura mater from trepan-cutting. Furthermore, a meticulous preoperative observation of exact relations among SAP and the structures around it on MRI and three-dimensional CT scan also ensured a safe and efficient resection. Expandable cage is convenient in implantation and controllable for the management of extension degree in lumbar spine surgery [21]. A previous study has reported percutaneous endoscopic lumbar interbody fusion technique with intervertebral cages using a titanium implant and an absorbable calcium phosphate substitute for the treatment of degenerative disc disease; the complication was rate up to 36%, while 13 in 57 cases appeared symptomatic cage migration [16]. In the process of PE-TLIF, a titanium expandable cage was used (see Figure 4). It has a good elasticity modulus and is parallelly expanded to have a good surface-to-surface contact with endplate, and it has a large adjustable range from 8mm to 13mm. The Sawtooth design on the top and bottom surface could avoid displacement after implantation. In our cases, expandable cages provided an instant stability of lumbar spine, and intervertebral space height was restored high enough to offer an indirect decompression of confined lateral recess. Until now, neither cage migration nor cage-related complications were found. During the operation, all the implantations were inserted percutaneously and manipulations were performed through cannulas in the procedure of PE-TLIF. This made merely a few injuries to paraspinal muscle, and posterior ligamentous complex remained intact so that stability of spine was fully preserved despite partial removal of SAP. In addition, damage to nerve structures is among the most serious complications of spinal surgery. The rate of intraoperative nerve injury in the literature was zero to 7% during conventional instrumented PLIF/TLIF, while reported incidence of dural tear ranged from 2% to 14%. However, no damages to the nerve structure have been found till now in our study [22]. We believed that depth-restricted guided SAP resection, meticulous preoperative observation, and the use of implantation tube with fork-shaped tip and that nerve root and dura mater had been carefully probed and protected before each step that might pose a threat to them by fluoroscopy and endoscopic visualization were the keys to avoid nerve injury during PE-TLIF. Although we have got a good clinical result, there were some complications that happened. One case had anterior annulus fibrosus that ruptured during endplate preparation and one case experienced temporary knee tendon hyperreflexia. Both complications happened on the early stage of performing PE-TLIF. We considered that the rupture might be due to the violent operation when scraping endplate using reamers, because this is the first surgery for endplate preparation under cannulas and surgeon needs time to be familiar with the operation. Yet, the cause for postoperative transient knee tendon hyperreflexia and the rapid recovery is still unknown.

In our experience, some important points should be paid attention to in the treatment of LSS by PE-TLIF. (1) Carefully preoperative plan should be made to avoid potential damage to important structures and set an individualized treatment for each patient. (2) Sufficient SAP resection was necessary to enlarge foramen and enable instruments with larger diameter to operate easily. (3) Particular attention should be paid when we are going to operate around nerve structures using fluoroscopy and endoscopic visualization. (4) We should manipulate gently during the whole course.

Up until now, there are few studies that reported endoscopic lumbar interbody fusion, and this is the first study about endoscopic lumbar interbody fusion using expandable cage and endplate preparation through cannulas. The major advantage of our study is that we innovatively develop some equipment such as guided SAP resection device and parallel expandable cage and improved the diameter of instruments to adapt to our nerve protection and percutaneous surgery concept. And all the operations were performed by the same orthopedic surgeon, which can avoid the differences caused by different surgeons' preference and experience. A number of data on the characteristics of patients, clinical results, and complications were included in our study. However, our study has its limitations. It is in fact a retrospective study and the number of patients is relatively small, and there is no control group to compare our results to. More prospective randomized controlled trials are needed to overcome the limitations of our study.

## 7. Conclusions

Our study suggested that percutaneous endoscopic transforaminal lumbar interbody fusion could acquire satisfactory treatment effects for patients with LSS, even for the patient who could not afford a general anesthesia. PE-TLIF will be a good alternative for the treatment of degenerative lumbar diseases in the near future.

## Figures and Tables

**Table 1:** Characteristics of 7 patients with PE-TLIF.

Case number	Sex	Age (years)	Pathological cause	Lesion segment	Intermittent claudication (meters)	Duration of symptom (years)	Comorbidity
1	Female	77	Severe LSS	L4/5	100	4	Pulmonary Fibrosis, Hypertension, Hyperlipidemia
2	Male	43	Moderate LSS	L4/5	None	3	None
3	Female	57	Moderate LSS	L4/5	100	2	None
4	Female	48	Mild LSS	L4/5	50	1	None
5	Female	43	Moderate LSS	L4/5	100	3	Hypertension, Diabetes
6	Female	68	LSS with degenerative spondylolisthesis	L4/5	100	20	None
7	Female	63	Moderate LSS	L4/5	None	2	None

**Table 2:** Results of treatment in the 7 patients.

Case number	Blood loss	Surgery time	Time to Ambulation	Time to Discharge	Fusion time	Follow-up	VAS (back)			VAS (leg)			ODI		Patient satisfaction
							Pre-operation	Post-operation	Final follow-up	Pre-operation	Post-operation	Final follow-up	Pre-operation	Final follow-up	
	(ml)	(hours)	(hours)	(days)	(months)	(months)									
1	140	280	48	6	6	21	8	3	1	6	2	1	56%	18%	good
2	150	270	24	4	6	17	8	2	0	5	2	1	40%	10%	excellent
3	50	320	24	3	6	17	8	2	1	9	2	0	38%	5%	excellent
4	100	270	20	3	3	14	7	2	1	6	1	1	62%	12%	excellent
5	50	290	18	3	6	12	6	2	0	8	1	0	57%	14%	excellent
6	300	280	24	4	3	12	8	3	1	5	2	1	63%	26%	good
7	30	290	24	5	3	12	7	2	2	4	1	1	59%	24%	good

## Data Availability

The data used to support the findings of this study are available from the corresponding authors upon request.
